# Translational outcomes relevant to neurodevelopmental disorders following early life exposure of rats to chlorpyrifos

**DOI:** 10.1186/s11689-020-09342-1

**Published:** 2020-12-16

**Authors:** Elizabeth L. Berg, Tianna M. Ching, Donald A. Bruun, Josef K. Rivera, Milo Careaga, Jacob Ellegood, Jason P. Lerch, Markus Wöhr, Pamela J. Lein, Jill L. Silverman

**Affiliations:** 1 MIND Institute and Department of Psychiatry and Behavioral Sciences, University of California Davis School of Medicine, Sacramento, CA USA; 2 MIND Institute and Department of Molecular Biosciences, University of California Davis School of Veterinary Medicine, Davis, CA USA; 3 Mouse Imaging Centre, Toronto Centre for Phenogenomics, The Hospital for Sick Children, Toronto, Ontario Canada; 4 Wellcome Centre for Integrative Neuroimaging, The University of Oxford, Oxford, UK; 5 Behavioral Neuroscience, Experimental and Biological Psychology, Philipps University of Marburg, Marburg, Germany; 6 Laboratory for Behavioral Neuroscience, Department of Biology, Faculty of Science, University of Southern Denmark, Campusvej 55, DK-5230 Odense M, Denmark

**Keywords:** Animal models, Behavior, Autism, Neurodevelopment, Toxicology, Pesticides, Chlorpyrifos, Imaging, Social, Rat, Vocalization, USV

## Abstract

**Background:**

Neurodevelopmental disorders (NDDs), including intellectual disability, attention deficit hyperactivity disorder (ADHD), and autism spectrum disorder (ASD), are pervasive, lifelong disorders for which pharmacological interventions are not readily available. Substantial increases in the prevalence of NDDs over a relatively short period may not be attributed solely to genetic factors and/or improved diagnostic criteria. There is now a consensus that multiple genetic loci combined with environmental risk factors during critical periods of neurodevelopment influence NDD susceptibility and symptom severity. Organophosphorus (OP) pesticides have been identified as potential environmental risk factors. Epidemiological studies suggest that children exposed prenatally to the OP pesticide chlorpyrifos (CPF) have significant mental and motor delays and strong positive associations for the development of a clinical diagnosis of intellectual delay or disability, ADHD, or ASD.

**Methods:**

We tested the hypothesis that developmental CPF exposure impairs behavior relevant to NDD phenotypes (i.e., deficits in social communication and repetitive, restricted behavior). Male and female rat pups were exposed to CPF at 0.1, 0.3, or 1.0 mg/kg (s.c.) from postnatal days 1-4.

**Results:**

These CPF doses did not significantly inhibit acetylcholinesterase activity in the blood or brain but significantly impaired pup ultrasonic vocalizations (USV) in both sexes. Social communication in juveniles via positive affiliative 50-kHz USV playback was absent in females exposed to CPF at 0.3 mg/kg and 1.0 mg/kg. In contrast, this CPF exposure paradigm had no significant effect on gross locomotor abilities or contextual and cued fear memory. Ex vivo magnetic resonance imaging largely found no differences between the CPF-exposed rats and the corresponding vehicle controls using strict false discovery correction; however, there were interesting trends in females in the 0.3 mg/kg dose group*.*

**Conclusions:**

This work generated and characterized a rat model of developmental CPF exposure that exhibits adverse behavioral phenotypes resulting from perinatal exposures at levels that did not significantly inhibit acetylcholinesterase activity in the brain or blood. These data suggest that current regulations regarding safe levels of CPF need to be reconsidered.

**Supplementary Information:**

The online version contains supplementary material available at 10.1186/s11689-020-09342-1.

## Background

The wide use of insecticides has raised a significant concern due to possible health effects associated with exposure to these compounds [1–5]. Insecticides are used globally to control crop pests in agriculture, to reduce household pests, to reduce insect damage to lawns and golf courses, and as mosquito control agents [6–8]. Among the most widely used insecticides are the organophosphorus pesticides (OPs), which include chlorpyrifos, parathion, and diazinon [9, 10]. Prenatal exposure to OPs has been associated with abnormal psychomotor ability, deficits in working memory and intelligence quotient, and disrupted behaviors in children [8, 11–18]. Experimental studies have also demonstrated an association between prenatal exposure to OPs and abnormal developmental reflexes [19].

The most extensively studied OP pesticide to date with respect to neurodevelopmental insults has been chlorpyrifos (CPF). Eaton and colleagues published a comprehensive review that examined the large body of toxicological data and epidemiological information describing effects of CPF in humans, with an emphasis on its controversial adverse effects on neurodevelopment [7]. Subsequently, the UC Davis Childhood Autism Risks from Genetics and Environment (CHARGE) study [8, 18] reported significant associations between prenatal OP exposures and mental and motor delays and an increased risk of autism spectrum disorder (ASD). Studies of a separate cohort of children in New York City provided corroborating behavioral data and brain structural neuroimaging of children prenatally exposed to CPF, illustrating enlargement of various cortical regions and effects on underlying white matter [20]. A recent meta-analysis of the epidemiological data concluded that there is a positive association between CPF and neurodevelopmental disorders, which warranted further investigation of CPF developmental neurotoxicity [21].

Rodents exposed to relatively high, but subtoxic, doses of CPF during early life exhibit delayed development of psychomotor reflexes [22, 23], sexual-social behaviors [24, 25], and impaired cognitive performance [26–29] later in life. But whether developmental exposure to CPF at levels that do not significantly inhibit acetylcholinesterase (AChE) causes phenotypes of relevance to neurodevelopmental disorders remains unclear. The goal of this study was to generate and use a rat model of developmental exposure to CPF to study the effect of environmentally relevant levels of CPF on a range of behaviors in young animals, including social communication, Pavlovian learning and memory, anatomical phenotypes determined by magnetic resonance imaging, and sexually dimorphic effects on these outcomes. The exposure paradigm used in this study was based on the finding from the CHARGE study which showed that pesticides had the most significant effect on health outcomes when exposure occurred during the third trimester [8, 18].

Characterizing the developmental neurotoxicity of environmentally relevant CPF exposures is required for assessing the risk that CPF poses to the developing brain, and for developing policies to protect the developing brain from this risk. In 2017, the federal EPA administrator denied a widespread petition to ban CPF, which is currently being appealed and battled in litigation. Additionally, the California Department of Pesticide Regulation announced in early 2019 that it will cancel the registration that currently allows chlorpyrifos to be sold in California. Thus, we aimed to use our preclinical model system to further clarify links between CPF exposure and adverse neurodevelopmental outcomes. This knowledge of CPF developmental neurotoxicity is crucial for implementing protective policies and mechanisms for estimating whether low dose exposures, via food and water consumption, pose real threats to human health.

## Methods

### Materials

Chlorpyrifos (CPF; *o*,*o*-diethyl [*o*-3,5,6-trichloro-2-pyridinol] phosphorothionate; 99.5% purity) was purchased from Chem Service (West Chester, PA, USA) and used within 6 months of purchase with interim storage as recommended by the manufacturer. Solutions were made weekly in NEOBEE® M-5 oil vehicle (Spectrum Chemical, Gardena, CA, USA) at their final concentrations and stored in a polypropylene container in the dark at room temperature.

### Subjects

Male and female Sprague-Dawley rats were purchased from Envigo (Indianapolis, Indiana) to generate cohorts for testing. All procedures were approved by the Institutional Animal Care and Use Committee (IACUC) at the University of California Davis and were conducted in accordance with the National Institutes of Health Guide for the Care and Use of Laboratory Animals. All animals were housed in a temperature-controlled vivarium maintained on a 12:12 light-dark cycle. OP pesticides were not applied in the vivarium before or during the study. To identify individual subjects, pups were labeled on the back via permanent marker on postnatal day 1, which was reapplied daily. As fur developed, animals were identified via tail marks, which were coded to allow investigators to run and score behaviors blind to the experimental group.

#### Cohorts

One cohort of rats, which consisted of 58 rat pups from 9 litters, was tested for early life communication. Rat pups were exposed daily to CPF (1.0 or 3.0 mg/kg) or vehicle (Neobee Coconut Oil; Spectrum Chemical MFG Corp) via s.c. injection (2 mL/kg) with a 30 gauge Hamilton syringe on postnatal day (PND) 1-4. On PND 8, pups underwent isolation-induced ultrasonic vocalization (USV) collection. These data, summarized in Supplementary Fig. S1, were the basis for the decision to test doses lower than 1.0 mg/kg in a second cohort.

A second cohort of rats, which consisted of 2 males and 2 females from each of 25 litters, was analyzed for early life and juvenile behavioral effects as well as juvenile neuroanatomical effects of CPF exposure. Rat pups were exposed daily to CPF at 0.1, 0.3, or 1.0 mg/kg or to an equal volume of vehicle (Neobee Coconut Oil; Spectrum Chemical MFG Corp) via s.c. injection (2 mL/kg) with a 30 gauge Hamilton syringe on PND 1-4. Litters were reduced to 8 pups (4 m and 4 f when possible) on PND 4, at which time, the culled littermates of the behavioral subjects were analyzed for acetylcholinesterase (AChE) activity in brain and blood at 1 h post-injection. The behavioral battery consisted of pup ultrasonic vocalizations and developmental milestones on PND 8, 12, and 16, locomotion in an open field, response to USV playback, and cued and contextual fear conditioning. After behavioral testing, brains were harvested and fixed for MRI analysis.

### Behavioral assays

#### Isolation-induced pup 40-kHz ultrasonic vocalizations

During the first few weeks of life, rodent pups emit ultrasonic vocalizations (USV) when separated from their mother and litter [30–32]. On PND 8, 12, and 16 pups were individually removed from the nest in a random order and placed into an open-top plastic isolation container containing corncob bedding. USV were collected for 3 min with an ultrasonic microphone (Avisoft Bioacoustics, Glienicke, Germany) using methods outlined previously [33, 34]. Immediately following USV collection, body temperature and body weight were measured.

#### Open field locomotion

Sedation or hyperactivity may have confounding effects on assays of sociability. Therefore, on PND 19, exploratory activity in a novel open field was automatically measured for 30 min as described previously [33, 34].

#### USV playback

Behavioral responses to playback of 50-kHz ultrasonic vocalizations were measured on PND 24-27 as previously described [34]. Briefly, rats were placed individually on an 8-arm elevated radial maze and presented with pro-social 50-kHz USV and a time- and amplitude-matched white noise acoustic stimulus control using an ultrasonic speaker (Avisoft Bioacoustics, Glienicke, Germany). Social exploratory and approach behavior in response to the USV were assessed, as was the behavioral response to the white noise stimulus.

#### Cued and contextual fear conditioning

Learning and memory were assessed on PND 30-33 using a previously described 3-day cued and contextual fear conditioning assay [35]. On day one, rats were trained to associate a foot shock with a specific environmental context as well as with a white noise auditory cue using automated chambers (Med Associates, Inc., St. Albans, Vermont). Approximately 24 h later, rats were re-exposed to the same context without the auditory cue and time spent freezing was quantified to assess contextual fear memory. Approximately 48 h following the initial training, rats were re-exposed to the auditory cue in a novel environmental context and time spent freezing was quantified to assess cued fear memory.

#### Ex vivo neuroimaging via magnetic resonance imaging

On PND 35-36, brains were flushed via transcardial perfusion (flow rate of 2 mL/min) with 50 mL phosphate-buffered saline (PBS) containing 10 U/mL heparin and 2 mM ProHance (a gadolinium-based contrast agent; Bracco Diagnostics Inc.), fixed with 50 mL 4% paraformaldehyde (PFA) in PBS containing 2 mM ProHance, and collected for neuroimaging following previously published protocols [36]. Following perfusion, brains were incubated in the 4% PFA solution for 24 h at 4 °C then transferred to a storage PBS solution containing 0.02% sodium azide. Brains were incubated in the storage solution at 4 °C for at least 1 month prior to scanning. Images were acquired and analyzed following a protocol previously described [33, 37]. Multiple comparisons were controlled for using the false discovery rate (FDR) with the significance level for the FDR-adjusted *p* value (*q*) set at *q* < 0.05 [38].

### AChE activity assay

One hour following the final CPF dosing on PND 4, pups were euthanized by decapitation and blood was collected by cardiac puncture into tubes containing EDTA as an anti-coagulant (Becton-Dickinson, Franklin Lakes, NJ). Blood was diluted 1:25 with phosphate buffer with 0.03% Triton X-100 (Fisher Scientific, Pittsburg, PA), vortexed, and snap frozen for later analysis. Brains were collected and snap frozen for later analysis. For the AChE activity assay, brain tissue was thawed on ice, homogenized in phosphate buffer with 1% Triton X-100, and AChE activity quantified using the standard Ellman Assay [39] with 5,5′-dithio-bis-2-nitrobenzoic acid (DTMB) and acetylthiocholine iodide (ASChI) as the substrates (Sigma-Aldrich, St. Louis, MO). Tetraisopropyl pyrophosphoramide (Sigma) was included to inhibit pseudocholinesterase. Blood AChE activity was normalized to hemoglobin levels, which were determined using a StanBio Laboratory Stat-Site M hemoglobin meter and test strips (Boerne, TX, USA). Brain AChE activity was normalized to protein concentration as determined using the BCA assay kit (Pierce, Rockford, IL).

### Statistical analyses

Developmental vocalizations, temperature, weight, and open field metrics were analyzed via repeated measures ANOVA with dose as the between-group factor and time as the within-group factor. Following detection of a significant main effect and/or time by dose interaction, *post hoc* testing was carried out using Holm-Sidak’s multiple comparisons test. Paired *t* tests (one per dose group) were used to compare time spent on the proximal and distal arms during the USV playback paradigm and locomotion during the playback test was compared using repeated measures or one-way ANOVA. Comparisons between freezing times were carried out for each test phase with one-way ANOVA. Acetylcholinesterase activity was analyzed using one-way ANOVA. Data were analyzed via GraphPad Prism. All significance levels were set at *p* < 0.05 and all *t* tests were two tailed. Multiple comparisons were corrected for via *post hoc* testing using Holm-Sidak’s multiple comparisons test.

## Results

### Developmental CPF exposure reduced isolation-induced pup ultrasonic vocalizations

**Fig. 1:**
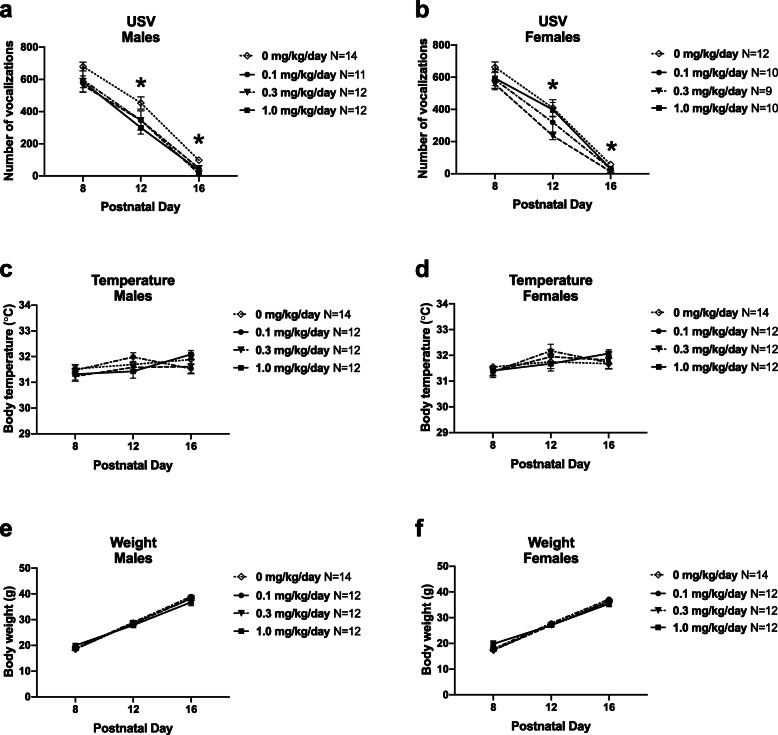
Early life CPF exposure reduces USV emission in male and female rat pups in a dose- and time-dependent manner. **a** Male pups exposed to 1.0 mg/kg/day CPF emitted fewer USV compared to vehicle controls on PND 12. By PND 16, all three male CPF exposure groups had significantly lower USV emission than controls. **b** In females, exposure to 0.3 mg/kg/day CPF led to reduced pup USV emission on PND 12 and 16. **c**, **d** Body temperature and **e**, **f** body weight immediately following USV collection were similar across exposure groups, eliminating these two variables as potential confounds on call quantity. Data are mean ± S.E.M. *p < 0.05, repeated measures ANOVA, Holm-Sidak’s multiple comparisons test *post hoc*

Pup ultrasonic vocalizations (USV) of infant rats measure an early communicative behavior between pups and mother. Isolation-induced USV were collected for 3 min as social communication signals in rat pups, as previously described [33]. CPF-exposed pups emitted significantly fewer USV across early development (Fig. 1a (males) *F*_(2, 90)_ = 286.5, *p* < 0.001; Fig. 1b (females) *F*_(2, 90)_ = 267.7, *p* < 0.001). As pups grow, they learn to temperature regulate, open their eyes, and are less reliant on maternal care, which is why USV decrease in number over developmental days. There was a significant main effect of experimental group on USV emission (*F*_males (3, 45)_ = 3.048, *p* < 0.05). Holm-Sidak *post hoc* analysis corrected for multiple comparisons highlighted significant differences on PND 12, when fewer USV were emitted in the 1.0 mg/kg CPF-exposed male pups, and on PND 16 in all CPF dose groups compared to vehicle. CPF-exposed female pups also emitted significantly fewer USV (*F*_(3, 37)_ = 2.949, *p* < 0.05). Holm-Sidak *post hoc* analysis highlighted strong trending differences on PND 8, as fewer USV were emitted in the 0.3 mg/kg CPF-exposed female pups (*p* = 0.061), and significant differences at PND 12 and 16 in the 0.3 mg/kg CPF-exposed female pups compared to vehicle.

Body weight and temperature were also collected to assure the reduced USV were not the result of being physically smaller as body weight is known to alter pup USV emission [30, 31]. Body temperature did not differ between CPF exposure groups and vehicle (Fig. 1c (males) *F*_(3, 46)_ = 0.5381, *p* > 0.05; Fig. 1d (females) *F*_(3, 46)_ = 0.67, *p* > 0.05). Weight did not differ between CPF exposure groups and vehicle (Fig. 1e (males) *F*_(3, 46)_ = 0.2745, *p* > 0.05; Fig. 1f (females) *F*_(3, 46)_ = 0.5234, *p* > 0.05), indicating typical growth and ability to thrive. In addition to being important control metrics for the pup USV assay, the observation that overall growth and health was not impacted by CPF exposure confirms the lack of systemic toxicity that has been reported with higher CPF doses using a functional observation battery [40, 41].

Analysis of typical early neurological reflexes did not reveal any significant differences between CPF-exposed pups and vehicle controls (Supplementary Fig. S2). Specifically, there were no significant differences between exposure groups in latencies to navigate upright in negative geotaxis and circle traverse, simple metrics for motoric, postural, and proprioceptive processes that underlie the ability of infant rodents to navigate on an inclined plane or to the outer rim from the center of circle (Fig. S2A (males) *F*_(3, 46)_ = 0.4776, *p* > 0.05; Fig. S2B (females) *F*_(3, 46)_ = 1.098, *p* > 0.05; Fig. S2C (males) *F*_(3, 46)_ = 1.224, *p* > 0.05; Fig. S2D (females) *F*_(3, 46)_ = 1.1319, *p* > 0.05).

### Normal locomotion and exploratory activity following developmental CPF exposure

**Fig. 2:**
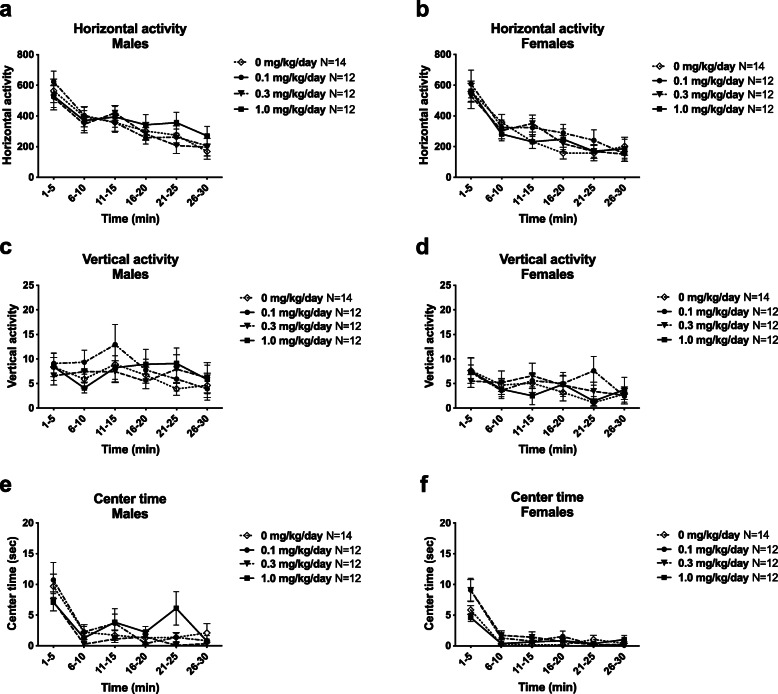
Early life exposure to CPF did not affect gross locomotor abilities. Both male and female rats of all exposure groups exhibited normal levels of (**a**, **b**) horizontal activity, (**c**, **d**) vertical activity, and (**e**, **f**) center time on PND 19. Data are mean ± S.E.M

Normal motor function following early life exposure to low doses of CPF was confirmed by lack of an effect of CPF on motor abilities in the open field exploratory locomotion task across a 30-min session. No CPF effect was observed in activity metrics of horizontal activity (Fig. 2a (males) *F*_(3, 46)_ = 0.2303, *p* > 0.05; Fig. 2b (females) *F*_(3, 46)_ = 0.3341, *p* > 0.05), vertical activity (Fig. 2c (males) *F*_(3, 46)_ = 0.2278, *p* > 0.05; Fig. 2d (females) *F*_(3, 46)_ = 0.2562, *p* > 0.05), or time spent in the center of the arena (Fig. 2e (males) *F*_(3, 46)_ = 0.7749, *p* > 0.05; Fig. 2f (females) *F*_(3, 46)_ = 2.150, *p* > 0.05).

### Reduced social exploration to affiliative 50-kHz ultrasonic calls (USV) in female CPF-exposed juveniles

**Fig. 3:**
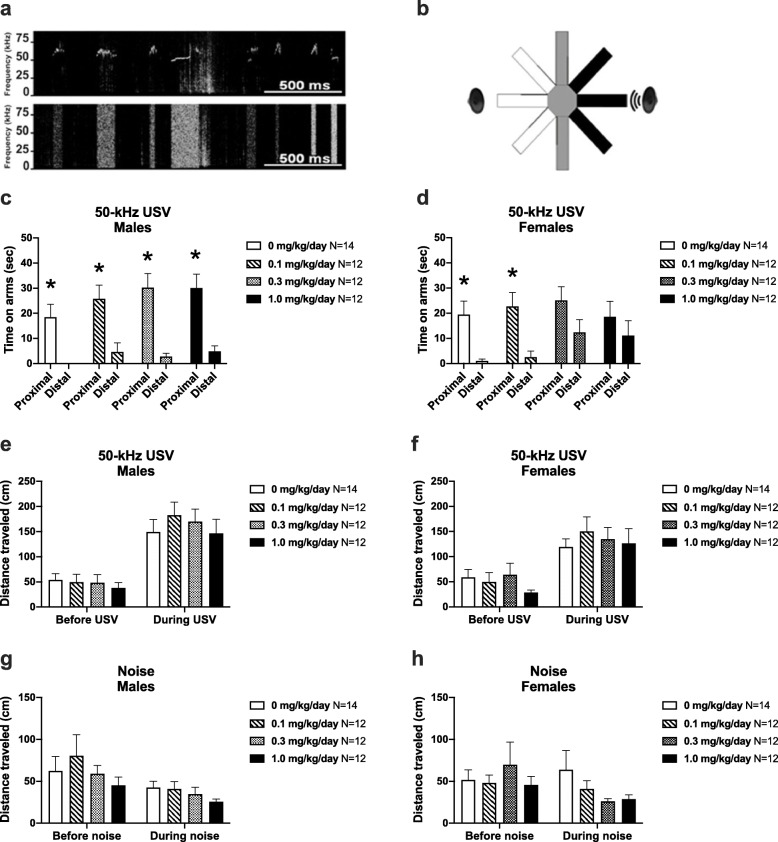
Lack of social approach to pro-social 50-kHz USV in female CPF-exposed rats. **a** Exemplary spectrograms showing 2 s of the pro-social 50-kHz USV (upper panel) and time- and amplitude-matched white noise (lower panel) stimuli used in the playback assay. **b** Illustration of the radial maze used, with arms proximal to the active ultrasonic speaker shown in black, arms distal shown in white, and neutral arms shown in gray. **c** During the minute of USV playback, males of all exposure groups spent significantly more time on the arms proximal to the speaker compared to the distal arms. **d** In females, only the vehicle and 0.1 mg/kg/day CPF groups showed a significant preference for the proximal arms. Female rats exposed to 0.3 mg/kg/day or 1.0 mg/kg/day did not spend significantly more time on the proximal arms compared to the distal arms. Regardless of exposure, (**e**) all males and (**f**) females displayed similar patterns of locomotion in response to playback of 50-kHz USV. **g** All males and (**h**) females exhibited comparable levels of movement during the minute before and the minute of white noise. Data are mean + S.E.M. **c**, **d**: *p < 0.05, paired *t* test, proximal vs. distal

Social exploratory behavior displayed by the male (Fig. 3c *t*_(1, 13)_ = 3.576, *p* < 0.005) and female vehicle control groups (Fig. 3d *t*_(1, 13)_ = 3.509, *p* < 0.005) was directed toward playback of pro-social 50-kHz USV, as reflected in the parameter of time spent on the arms proximal to the sound source emitting 50-kHz USV as compared to the distal arms of the radial maze. All groups of male juvenile rats (vehicle and each dose of CPF) spent significantly longer on the arms proximal to the speaker emitting the 50-kHz USV upon playback (Fig. 3c (0.1 dose) *t*_(1, 13)_ = 2.738, *p* < 0.02; Fig. 3c (0.3 dose) *t*_(1, 13)_ = 4.587, *p* < 0.001; Fig. 3c (1.0 dose) *t*_(1, 13)_ = 4.502, *p* < 0.001). In contrast, the 0.3 mg/kg and 1.0 mg/kg CPF-exposed females rats did not spend significantly more time on the proximal arms (Fig. 3d (0.1 dose) *t*_(1, 13)_ = 3.001, *p* < 0.005; Fig. 3d (0.3 dose) *t*_(1, 13)_ = 1.373, *p* > 0.05; Fig. 3d (1.0 dose) *t*_(1, 13)_ = 0.7127, *p* > 0.05).

All groups demonstrated a similar locomotor response to the 50-kHz USV, characterized by elevated movement during the USV as compared to baseline (Fig. 3e (males, time) *F*_(1, 46)_ = 100.5, *p *< 0.0001; Fig. 3e (males, group) *F *_(3, 46)_ = 0.337, *p *> 0.05; Fig. 3e (males, time x group) *F *_(3,  46)_ = 0.533, *p *> 0.05; Fig. 3f (females, time) *F*_(1, 46)_ = 45.90, *p *< 0.0001; Fig. 3f (females, group) *F*_(3, 46)_ = 0.379, *p *> 0.05; Fig. 3f (females, time x group) *F*_(3, 46)_ = 0.682, *p* > 0.05). Distance traveled in response to the white noise control stimulus did not differ between exposure groups, and all groups exhibited comparable levels of locomotion before (Fig. 3g (males) *F*_(3, 46)_ = 0.707, *p* > 0.05; Fig. 3h (females) *F*_(3, 46)_ = 0.448, *p *> 0.05) and during the noise stimulus (Fig. 3g (males) *F*_(3, 46)_ = 1.094, *p* > 0.05; Fig. 3h (females) *F*_(3, 46)_ = 1.596, *p* > 0.05). These findings rule out the possibility of a confounding hearing deficit in the CPF-exposed groups.

### CPF-exposed rats demonstrated intact contextual and cued fear memory

**Fig. 4:**
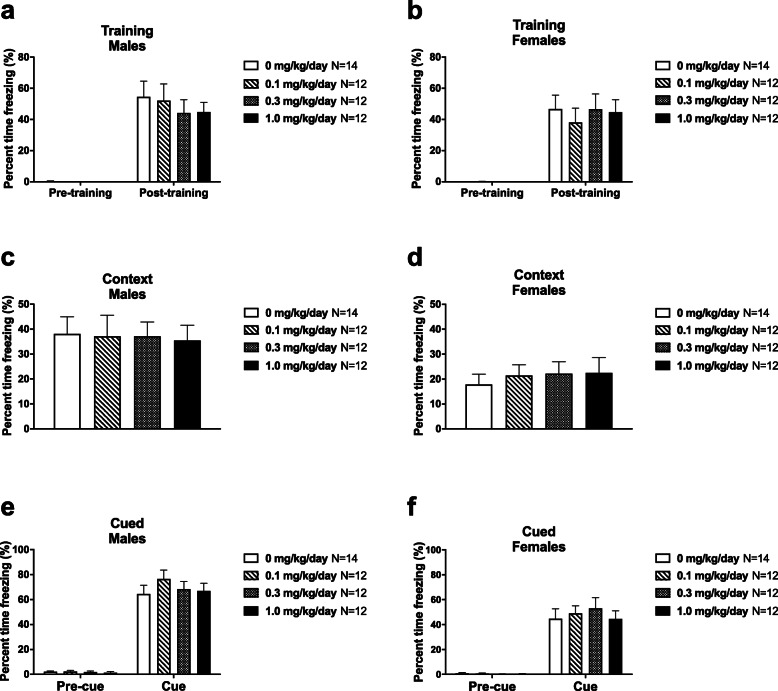
Intact contextual and cued fear memory in rat pups exposed to CPF during early life. (**a**) Male and (**b**) female rats of all exposure groups exhibited typical levels of freezing following foot-shock training, (**c**, **d**) in the same context 24 h later, and (**e**, **f**) upon hearing the auditory cue in a new context 48 h after training. Data are mean + S.E.M

Learning and memory was evaluated using two measures of Pavlovian fear conditioning with a 24 h contextual component and a 48 h tone cued fear conditioning. High levels of freezing were observed subsequent to the conditioned stimulus (CS)—unconditioned stimulus (UCS) pairings on the training day, in both exposed groups (Fig. 4a (males) no group difference in post-training freeze scores, *F*_(3, 46)_ = 0.3342, *p* > 0.05; Fig. 4b (females) no group difference in post-training freeze scores, *F*_(3, 46)_ = 0.2033, *p* > 0.05), indicating no confounds and no deficits in the learning of the associations between the context stimuli and tone cues. No exposure group difference in freezing was observed 24 h following CS-UCS training (Fig. 4c (males) *F*_(3, 46)_ = 0.02571, *p* > 0.05; Fig. 4d (females) *F*_(3, 46)_ = 0.2045, *p* > 0.05) when placed in the context chamber from conditioning training with identical stimulus cues. Levels of freezing, pre- and post-cue presentation 48 h after training, showed no effect of exposure (Fig. 4e (males, pre-cue) *F*_(3, 46)_ = 0.1365, *p* > 0.05; Fig. 4e (males, cue) *F*_(3, 46)_ = 0.6103, *p* > 0.05; Fig. 4f (females, pre-cue) *F*_(3, 46)_ = 0.3858, *p* > 0.05; Fig. 4f (females, cue) *F*_(3, 46)_ = 0.2999, *p* > 0.05).

### Neuroanatomical pathology at PND 35 following developmental CPF exposure

**Fig. 5:**
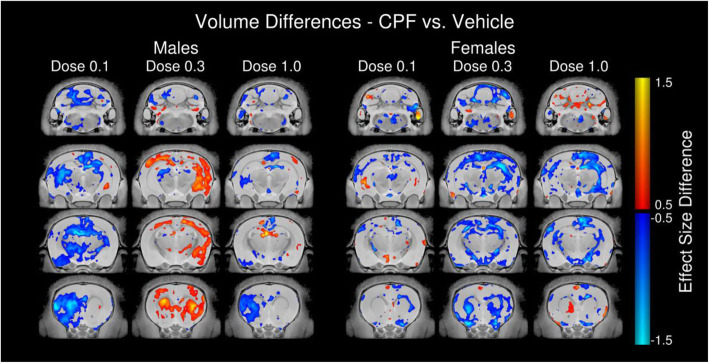
Neuroanatomical pathology at PND 35 in rats exposed to CPF during early life. **a** Representative coronal slice series for males and females highlighting effect size differences in absolute brain volume (mm^3^) between vehicle and 0.1 mg/kg/day, 0.3 mg/kg/day, and 1.0 mg/kg/day CPF exposure groups. Red-to-yellow coloration indicates areas that trended larger in CPF-exposed groups compared to vehicle and dark-to-light blue coloration indicates areas that were smaller in CPF-exposed groups compared to vehicle

Overall, the total brain volumes were not observed to be different between groups (1683 ± 101 mm^3^ for vehicle, 1649 ± 51 mm^3^ for a CPF dosage of 0.1 mg/kg, 1675 ± 123 mm^3^ for 0.3 mg/kg, and 1662 ± 68 mm^3^ for 1.0 mg/kg). A difference in total brain volume between vehicle and CPF exposure at 0.3 mg/kg of −2.27% observed in the females was a mere one hundredth from significance (*p* = 0.06, *q* = 0.22). There were no significant findings for any CPF exposure group nor for any sex when correcting for multiple comparisons. There was a trend toward a decrease in the hippocampal region (−3.29%, *p* = 0.03, *q* = 0.22), which appeared to be localized to Ammon’s Horn (−3.52%, *p* = 0.02, *q* = 0.22). Additional trends toward a loss in volume were found in the fiber tracts (−2.61%, *p* = 0.03, *q* = 0.22), with the strongest trends found in the fimbria (−3.63%, *p* = 0.02, *q* = 0.22) and the cortical spinal tract (−5.11%, *p* = 0.01, *q* = 0.22). Voxelwise comparisons also revealed no significant differences, but again interesting trends were seen in the female rats exposed to CPF at 0.3 mg/kg (Fig. 5). Interestingly, at the 0.3 mg/kg dosage, opposite effects are seen in males versus females with males showing positive effect size differences and females showing negative effect size differences (Fig. 5).

### Normal brain and blood AChE activity following CPF exposure

**Fig. 6:**
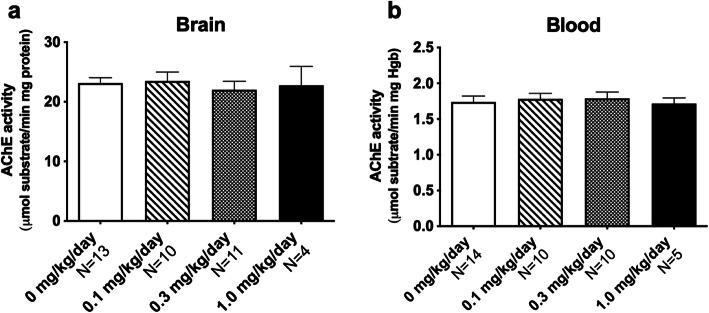
Developmental CPF exposure did not significantly inhibit acetylcholinesterase (AChE). **a** Regardless of exposure group, all pups exposed to CPF on PND 1-4 showed normal AChE activity in the (**a**) brain and (**b**) blood at 1 h following the final dose on PND 4. Data include males and females and are mean + S.E.M

None of the three doses of CPF significantly altered the enzymatic activity of AChE in the brain (Fig. 6a *F*_(3,35)_ = 0.1252, *p* > 0.05) or in the blood (Fig. 6b *F*_(3,34)_ = 0.2137, *p* > 0.05).

## Discussion

There is an extensive literature describing the developmental neurotoxicity of the OP pesticide chlorpyrifos (CPF). Epidemiological studies [8, 11–17], which provide compelling links between early life exposure to OPs and abnormal early cognitive development, may offer insights into the rising prevalence of neurodevelopmental disorders (NDDs). Epidemiological studies suggest that prenatal exposure to CPF, particularly during the second or third trimester, is associated with significant mental and motor delays and with a clinical diagnosis of NDD, including ADHD and ASD [8, 16, 18, 20, 42]. To date, there have been fewer reports in preclinical mouse and rat models testing the hypothesis that developmental CPF exposure impairs behaviors relevant to the broad NDD phenotype. Herein, we report the initial behavioral and anatomical characterization of a rat model of developmental CPF exposure at doses that do not significantly inhibit acetylcholinesterase (AChE) activity. The most significant effect, reduced ultrasonic vocalization emission in pups, was observed in both sexes. We also discovered reduced social communication via a 50-kHz USV playback assay, a USV call and behavioral response task that can only be performed/observed in rats, which supports our hypothesis because aberrant social communication aligns with the clinical profiles of many NDDs. Structural imaging illustrated a large number of changes in brain volume and a variety of neuroanatomical phenotypes. Collectively, this study identified unique NDD-relevant functional and anatomical phenotypes as preclinical outcomes in response to developmental CPF exposures that had no effects on AChE activity.

This is the first report of reduced ultrasonic vocalizations in rat pups following developmental CPF exposure. Ultrasonic vocalizations in pups are crucial signals that elicit maternal care, without which pups would not be able to thermoregulate or suckle [43]. Reduced USV communication has been discovered in many genetic rat models of NDD, including those with mutations in synaptic genes, such as *Shank3*, cellular housekeeping genes such as ubiquitin ligase *Ube3a* that causes Angelman syndrome, and the calcium channel gene *Cacna1c* [33, 34, 44], as well as numerous genetic mouse models of NDD, including 16p11.2 deletion syndrome [45], the Ca(V)1.2 L-type calcium channel gene that causes Timothy Syndrome [46], synaptic genes such as neuroligins [47], and high confidence ASD candidate genes, such as *Tbx1* [48]. Reduced USV communication has also been reported in models of environmentally induced NDD phenotypes, including maternal immune activation [49], prenatal exposure to valproic acid [50, 51], and developmental exposure to traffic-related air pollution [35].

While we exposed rats to CPF during the first days of postnatal life, our findings are consistent with earlier literature showing that exposure to CPF during the gestational period resulted in altered behavioral and physical development in rodent pups in a sex-dependent manner. Venerosi and colleagues reported delayed somatic growth, reduced ultrasonic vocalizations, and increased latency to emit calls in male and female CD-1 mouse pups prenatally exposed to CPF [52], corroborating clinical reports in epidemiological studies [53]. Among mice exposed to the subtoxic doses of 1 and 3 mg/kg/d CPF on PND 1-4 and PND 11-14, hyperactivity was observed only in those exposed to 3 mg/kg/d CPF on PND 11-14 [24], which is consistent with our observation that the lower CPF doses tested in this study caused neither hypoactivity nor hyperactivity in rats in the open field task. However, in contrast to our findings, in the CD-1 strain mouse studies, the PND 1-4 exposure reduced brain cholinesterase activity by 25%. Studies of rats injected with 1 mg/kg/d CPF on PND 1-4 have also reported significantly reduced AChE activity in the brain ranging from 20 to 60% depending on sex and the interval between the last injection of CPF and the collection of tissue for analyses [22, 54]. The key differences between our study and the two earlier rat studies was the vehicle used for CPF dosing: we used a coconut oil preparation whereas the previously published rat studies, and the mouse study, used DMSO. Pharmacokinetic studies in adult rats have shown that subcutaneous administration of CPF in corn oil resulted in faster absorption and metabolism of CPF compared to a subcutaneous administration of CPF in DMSO [55]. Whether this is the reason why we did not see a significant inhibition of AChE whereas other studies have despite using the same doses over the same developmental ages in the same rat strain has yet to be determined.

Exposure to CPF at 1 mg/kg during early postnatal life elicited deficits in reflex righting and geotaxis behavior in female rat pups [22]. This was also observed recently in Harlan-derived B6 mice exposed to 2.5 mg/kg of CPF on gestational days 12-15 [23]. Exposure to CPF at 1.5 mg/kg in early postnatal life reduced body weight in male Sprague-Dawley rats [28]. In contrast, we observed no effect of the CPF doses on the neonatal reflexes of negative geotaxis and circle traverse.

We discovered impaired juvenile behavioral responses to the playback of 50-kHz USV, a positive affiliative social contact call associated with play and social interactions. Reductions in playback social approach have been observed in other genetic rat models of NDD such as *Shank3*, *Ube3a*, and *Cacna1c* [33, 34, 44]. Juvenile social approach during playback is a bidirectional social communication behavior commonly studied in rats rather than mice as most reports that use choice playback in mice use sexual mating calls to elicit behavior [56, 57] and because inbred or congenic B6J mice cannot hear in the frequency range of ultrasonic vocalizations [58, 59]. We observed that female rats exposed to CPF at 0.3 or 1.0 mg/kg have a deficit in the key social approach behavior following a playful 50-kHz USV. This effect is not a consequence of deficits in psychomotor activation, motor abilities, or hearing. This conclusion is based on no evidence of motor impairments in the open field, pre-training or pre-cue activity in fear conditioning, and the total distance traversed following presentation of 50-kHz USV, a key control metric for the social playback assay. These observations suggest the arousal-evoking component of the playback is intact but that the deficit is specific to the social approach parameter of the assay. This could be due to multiple reasons including the CPF-exposed rats not being able to localize the sound source, the CPF-exposed rats having less dopaminergic-mediated motivation for social reward, and/or the CPF-exposed rats not being able to understand the communicative function of the pro-social 50-kHz USV. Differentiating between the various possible explanations will require future experiments beyond the scope of this initial generation of the rat model [60–62].

Our observations of the effects of developmental CPF exposure are novel because they are among the first reports of NDD-relevant phenotypes in a socially sophisticated rodent species, the rat. Our findings extend earlier literature of unusual social behavior elicited by developmental CPF exposure in mouse models with a wide variety of doses and exposure windows. Mouse research showed that neonatal CPF exposure (3 mg/kg) increased sexual social soliciting behaviors, specifically aggressive behaviors in mice exposed to a subtoxic dose of CPF during a different early life period (PND 11-14) [24]. Adult male mice exposed to CPF prenatally (6 mg/kg) or postnatally (3 mg/kg) exhibited increased aggressive behaviors during a social dyadic interaction test [25]. The increase in aggressive behaviors in male mice at an age when affiliative behaviors should be prevalent suggests a deviation from the species-typical pattern of social behavior [63]. Moreover, gestational and neonatal exposure to CPF resulted in impaired nest building and maternal aggression in lactating female mice, indicating impaired maternal behavior [64, 65]. Mounting evidence suggests that CPF could disrupt the endocrine system and adversely affect social behavior in a sexually dimorphic manner, as extensively reviewed elsewhere [66]. A recent study reported reductions in social preference ratio in Harlan-derived B6 mice of both sexes exposed to 2.5 mg/kg or 5.0 mg/kg of CPF on gestational days 12-15 [67]. Our findings contrast with a report of increased social play in juvenile rats using CPF doses of less than 1.0 mg/kg [68]; however, the dosing in our study was across PND 1-4 while that exposure paradigm started at PND 10 and lasted for 7 days, emphasizing the critical effect of timing in behavioral toxicology. Detailed examination of reciprocal social play interactions is planned for subsequent studies.

This is also the first report to utilize ex vivo MRI to examine broad effects of developmental CPF exposure. Neuroanatomically, the CPF dosages had no significant effects on the mesoscopic brain structure of the rats. There were interesting trends at the 0.3 mg/kg dose, in particular, the divergent direction of the structural findings in males (increased volume) and females (volumetric reductions). Typically, in neuroimaging studies of genetic mouse models, significant differences in relative regional volumes are usually found in about 65% of cases [69]. These types of studies are powered at 80% which should typically find regional differences in the mouse at 3-5%, which is consistent with some of the differences at 0.3 mg/kg dose. Powered at this level, our studies tend to be more sensitive to widespread changes rather than focal ones, which could explain our observation of “trends” in this work. Additionally, it is possible that there is increased variability in the rat versus mouse due to the substantially more variable genetic background than in the mouse, but this remains to be tested for CPF exposures specifically and is only beginning to be compared in genetic rat models of NDDs. It should also be noted that the findings here do not indicate that there are no structural differences due to CPF, only that no significant changes are detectable at the mesoscopic resolution of the MRI. Going forward, we will perform more regionally targeted neuroanatomy using the strongest trending areas observed in this study.

We did not observe effects of developmental CPF exposure on motor activity. While others have observed changes, those effects were found at higher dose exposures and/or in mice. For example, pre- and postnatal CPF exposure (6 or 3 mg/kg, respectively) markedly increased locomotor activity in adult male mice tested in the open field [25]. These results corroborated the finding of decreased habituation rate in rats exposed to 1.0 mg/kg CPF during the later postnatal period [29]. However, habituation rate on a radial maze is not the same metric as assessing locomotion in a novel arena. Exposure to CPF at 1 mg/kg during early postnatal life reduced locomotor activity and rearing in adolescent Sprague-Dawley male rats [22]. However, rats postnatally exposed to diazinon (0.5 and 2 mg/kg), another OP pesticide, exhibited normal locomotor activity and a normal habituation pattern in a 1 h figure-8 locomotor activity test [70]. Taken together, there is a lack of consistency and corroboration suggesting that locomotor activity is not a sensitive, reproducible, and rigorous endpoint for low-level OP exposures in preclinical rodent models. Adverse effects on performance are often observed in a single laboratory, and follow-up literature is unable to reproduce or delineate cognitive impairments from motoric dysfunction [71, 72]. Future directions intended to comprehensively assess motor behavior with a specific behavioral battery that includes gait, balance, coordination, velocity, temporal and spatial dynamic metrics over rudimentary activity, and/or habituation will lead to improved translational value. This will allow for direct comparisons to humans using devices such as pressure sensitive mats, electromyographic recordings, and wrist or ankle monitors that measure activity/balance.

We also did not observe any deficits in cued and contextual fear conditioning, a classic yet simplistic assay of learning and memory. Earlier studies found that juvenile rats exposed to doses of CPF (0.3 or 7.0 mg/kg) early in life (PND 7, 11, and 15) exhibited spatial learning deficits in the Morris water maze [27]. A second cohort of juvenile rats exposed to CPF (0.3 and 7.0 mg/kg) at a later age (PND 22 and 26) exhibited similar impairments [27]. Neonatal CPF exposure (5 mg/kg) on PND 1-4, but not on PND 5-11, impaired radial-arm maze choice accuracy during the initial phase of training when the test situation is novel or cholinergic inputs are required [29]. As these assays measure substantially different components of learning and memory, we are cautious to state there is a contrast between our findings and that of these earlier reports. As most literature points to a significant effect of CPF on learning and memory, we attribute differences between previous results and our lack of this finding in fear conditioning to the lack of task sensitivity as well as varying doses and timing of exposures. Future directions intend to comprehensively assess the adverse effects of developmental exposure to CPF on learning and memory with improved translational value by using computerized touchscreen technology, which will hopefully unify the current literature, as each earlier report measured a different parameter or form of learning and memory.

A key question is the relevance of the doses used in this study to human exposures. The estimated average daily combined intake of chlorpyrifos and chlorpyrifos-methyl for infants ranges from 0.003 μg/kg/day [73] to 0.018 ug/kg/day [7]. While these levels are many orders of magnitude lower than the doses administered to postnatal rats in this study, it is critical to remember that the human data reflect estimated average daily exposures and do not take into account exposures during periods of active pesticide exposure in the home, school, or nearby agricultural fields. A more relevant comparison is CPF levels in human cord blood at birth, which range from 3.7 pg/g [16] to > 6.17 pg/g [74]. The peak level of CPF in the blood of PND 5 rats dosed with CPF at 1 mg/kg in DMSO (s.c.) was approximately 9 ng/ml [75]. While these data imply that our dosing paradigm likely resulted in CPF levels in the postnatal rats that are 2-3 orders of magnitude higher than are detected in human neonates, direct comparison of these levels to determine relevance is complicated by the observation that rat blood contains high levels of circulating carboxyesterases, which metabolically inactivate organophosphorus (OP) insecticides, such as CPF, whereas humans have low levels of these enzymes [74]. Thus, the percentage of any CPF dose that reaches the brain is likely to be significantly lower in rats than in humans.

Biological mechanisms of OP toxicity are complex. The canonical mechanism of OP neurotoxicity is inhibition of AChE, which hydrolyzes acetylcholine. More importantly, and most relevant to the present work, it is widely posited that developmental OP neurotoxicity involves mechanisms other than or in addition to AChE inhibition, as recently reviewed [76, 77]. The robust behavioral findings reported in these animals exposed to CPF at doses that have no significant effect on blood or brain AChE clearly support non-cholinergic mechanisms as contributing to effects that are translationally relevant for NDDs. Further biochemical assays using this exposure paradigm must be evaluated in future follow-up studies.

## Conclusions

Collectively, our results indicate that early life exposure to the OP pesticide CPF leads to behavioral and some possible neuroanatomical differences in rats that are highly relevant to NDDs. Interestingly, the effects of CPF we observed were strong, observed at multiple timepoints of development, in both sexes, and at doses that did not inhibit AChE activity. By developing and utilizing a novel rat model of developmental CPF exposure, which leverages the sophisticated vocal communication system of rats, we characterized the effect of environmentally relevant CPF exposures on a range of behaviors and were able to detect impaired social communication in pups and juveniles. Critically, these effects occurred in the absence of AChE inhibition, which is the endpoint used to regulate OP exposures to protect human health.

The public health implications of these results are significant, as pesticides continue to be widely used resulting in widespread human exposures. With the laws regarding pesticide application currently under debate, this work provides timely and much needed experimental evidence to inform future policy decisions.

## Supplementary Information

**Additional file 1: Supplementary Fig S1**. Reduced USV emission in rat pups exposed to CPF during early life**. a** Male pups exposed to CPF emitted normal numbers of USV on PND 8 compared to vehicle controls while **b** exposure to 1.0 mg/kg/day CPF in females resulted in reduced USV emission relative to controls. Data are mean + S.E.M. *p < 0.05, one-way ANOVA, Holm-Sidak’s multiple comparisons *post hoc*. **Supplementary Fig S2**. No effect of early life CPF exposure on developmental milestone achievement. **a,b** Performance on the negative geotaxis and **c,d** circle traverse tasks did not differ between exposure groups for males or females, respectively. Data are mean ± S.E.M.

## Data Availability

Datasets available from corresponding author upon reasonable request.
