# Supplementary material for: Translational outcomes relevant to neurodevelopmental disorders following early life exposure of rats to chlorpyrifos
Source: J Neurodev Disord. 2020 Dec 16;12:40. doi: 10.1186/s11689-020-09342-1 (PMC7745485; doi:10.1186/s11689-020-09342-1)
Supplement: Supplementary file 1 — Additional file 1: Supplementary Fig S1. Reduced USV emission in rat pups exposed to CPF during early life. a Male pups exposed to CPF emitted normal numbers of USV on PND 8 compared to vehicle controls while b exposure to 1.0 mg/kg/day CPF in females resulted in reduced USV emission relative to controls. Data are mean + S.E.M. *p < 0.05, one-way ANOVA, Holm-Sidak’s multiple comparisons post hoc. Supplementary Fig S2. No effect of early life CPF exposure on developmental milestone achievement. a,b Performance on the negative geotaxis and c,d circle traverse tasks did not differ between exposure groups for males or females, respectively. Data are mean ± S.E.M. [file 11689_2020_9342_MOESM1_ESM.docx]

**Supplementary Fig S1. Reduced USV emission in rat pups exposed to CPF during early life.** **a** Male pups exposed to CPF emitted normal numbers of USV on PND 8 compared to vehicle controls while **b** exposure to 1.0 mg/kg/day CPF in females resulted in reduced USV emission relative to controls. Data are mean + S.E.M. **p* < 0.05, one-way ANOVA, Holm-Sidak’s multiple comparisons *post hoc*.

**Supplementary Fig S2. No effect of early life CPF exposure on developmental milestone achievement. a,b** Performance on the negative geotaxis and **c,d** circle traverse tasks did not differ between exposure groups for males or females, respectively. Data are mean ± S.E.M.
